# Evaluation of a Luffa Cylindrica-Based Biosorbent as an Alternative for Sample Preparation in the Multiresidue Determination of Pesticides in Fish Fillets by UHPLC-MS/MS

**DOI:** 10.3390/foods15203696

**Published:** 2026-10-17

**Authors:** Franciele Fátima Machado, José Dilson Francisco da Silva, Luana Floriano, Juliana Diniz de Barros Kuntz, Alcides Ferreira Coradini, Osmar Damian Prestes, Renato Zanella

**Affiliations:** 1 Chemistry Department, Federal University of Santa Maria (UFSM), Santa Maria 97105-900, RS, Brazil; francimachadote@gmail.com (F.F.M.); luanaflorianoqmc@gmail.com (L.F.); juukuntz30@gmail.com (J.D.d.B.K.); alcidesfcoradini@gmail.com (A.F.C.); osmar.prestes@ufsm.br (O.D.P.); 2 Federal Institute of Education, Science, and Technology of Tocantins, Araguaina 77824-838, TO, Brazil; dilson13@gmail.com

**Keywords:** fish, pesticides, food, biosorbent, QuEChERS, UHPLC-MS/MS

## Abstract

The bioaccumulation of pesticides in fish can pose risks to human health. In this study, a biosorbent based on chitosan-modified vegetable sponge (*Luffa cylindrica*) for use in the clean-up stage of extracts intended for the multiresidue determination of pesticides in fish fillets by ultra-high-performance liquid chromatography coupled with tandem mass spectrometry (UHPLC-MS/MS). The proposed biosorbent is a sustainable, renewable, and low-cost alternative for cleaning food extracts via dispersive solid-phase extraction (d-SPE). The method consisted of extracting the analytes using the original QuEChERS procedure, followed by a clean-up step using the developed biosorbent and subsequent determination by UHPLC-MS/MS. The efficiency of the clean-up, evaluated primarily by monitoring the removal of phospholipids, demonstrated a high capacity to remove co-extracted compounds from the matrix. The method was validated for 130 pesticides in fish fillets, with method limit of quantification (LOQ) of 0.001 mg kg^−1^ for most of the evaluated pesticides. Application in commercial fish fillet samples resulted in the detection of pesticides at concentrations between 0.0062 and 0.1308 mg kg^−1^, demonstrating that the biosorbent is a promising alternative for the preparation of samples from complex lipid matrices.

## 1. Introduction

The intensive use of pesticides in agriculture has raised concerns due to their potential impact on aquatic ecosystems and non-target organisms [1]. These compounds can enter aquatic environments through surface runoff, leaching, and drift during application, contributing to the contamination of various environmental compartments and aquatic organisms [2]. Exposure of fish to these contaminants, even at sublethal concentrations, can trigger oxidative stress, histopathological changes, and cellular damage in organs such as the gills and liver, compromising essential physiological functions [3,4]. In recent years, several analytical strategies have been developed for the determination of pesticides in fish, such as ultra-high-performance liquid chromatography coupled with tandem mass spectrometry (UHPLC-MS/MS) [5]. This approach allows for high sensitivity, selectivity, and the simultaneous quantification of a wide range of micropollutants, even at ultra-trace concentrations in complex fish matrices [6]. Among the main challenges associated with the analysis of pesticide residues in fish is the presence of high lipid levels, which are partially extracted during sample preparation, hindering the analyses and causing matrix effects (MEs) [7].

The QuEChERS method is considered particularly suitable and effective in the extraction of a wide range of pesticide residues from various classes, especially in foods of animal origin, including dairy products, meat, fish, and eggs, and has demonstrated satisfactory analytical performance, even given the high complexity of these matrices [8,9,10,11]. In line with trends in green analytical chemistry, the sustainability of the QuEChERS method has been widely recognized, as it significantly reduces the use of solvents and materials compared to traditional techniques, thereby decreasing the environmental impact associated with sample preparation [12].

Extraction and extract clean-up, such as using dispersive solid-phase extraction (d-SPE) in the QuEChERS methods, are widely used to remove lipid interferents [13,14]. This method consists of two steps: extraction/partitioning and extract clean-up. The extraction step uses acetonitrile as the extraction solvent, followed by the addition of anhydrous magnesium sulfate (MgSO_4_) to partition the organic solvent [15]. Compared to other traditional methods used for food sample preparation, QuEChERS is a simple, rapid, and efficient procedure that requires few steps and, because it is performed in closed tubes, reduces operator exposure to solvents [16]. During the extract clean-up step, sorbents such as primary-secondary amine (PSA), octadecylsilane (C18), and graphitized carbon (GCB) are generally used [17,18,19,20], including mixtures thereof, using dispersive solid-phase extraction (d-SPE), which simplifies this step. Extract clean-up is a critical step in the analysis of pesticide residues in complex matrices, since inadequate removal of co-extracted components can compromise ionization efficiency and the analytical response obtained by mass spectrometry [21]. The C18 sorbent has been used to purify extracts from samples with high lipid content due to its ability to retain nonpolar compounds, such as lipids [22]. Other specific commercial sorbents have also been used for this purpose, notably EMR-Lipid^®^, which selectively removes lipid co-extracts [23], and Z-Sep+^®^, composed of zirconium dioxide and silica, which exhibits high affinity for proteins and lipids [22].

However, in food matrices with high chemical complexity, the search for materials with greater selectivity and adsorption efficiency has driven the development of new sorbents, including multi-walled carbon nanotubes (MWCNTs), graphene oxide (GO), and metal-organic frameworks (MOFs) [24,25,26]. Despite the performance of these materials, limitations related to synthesis, cost, availability, and environmental impact are driving research into alternatives based on renewable resources. In this context, naturally derived sorbents have gained prominence as potential materials for sample preparation applications, combining clean-up efficiency with greater sustainability [27].

In this context, naturally derived biosorbents have been explored as promising alternatives for applications in the extract clean-up stage. The loofah (*Luffa cylindrica*) has gained prominence due to its three-dimensional porous structure, composed of an interconnected network of fibers that provides high structural stability and the potential for interaction with various organic compounds [28]. In addition, this material exhibits high adsorption capacity, especially for oily compounds, a characteristic that contributes to its application in processes involving the removal of interfering substances. Traditionally used as a cleaning material, including in agricultural applications, *Luffa cylindrica* stands out for its availability, low cost, and renewable origin [29]. However, despite the favorable properties of the loofah, chemical modifications can be employed to enhance its ability to interact with specific compounds. In this context, chitosan is a biopolymer that has attracted interest due to the presence of amino (–NH_2_) and hydroxyl (–OH) functional groups, which can establish different chemical interactions with molecules present in the matrix, including phospholipids [30]. Chitosan is a polysaccharide obtained by deacetylation of chitin, the main structural component of the exoskeletons of crustaceans, such as shrimp, crabs, and lobsters. In addition to its biocompatibility, biodegradability, and low toxicity, chitosan exhibits high affinity for various organic compounds, making it an effective material for purifying food extracts to determine pesticide residues in foods with high lipid content [31,32].

Given the potential of biosorbent materials for sample preparation applications, this study aimed to develop and evaluate a biosorbent produced from loofah (*Luffa cylindrica*) modified with chitosan for use in the clean-up stage of fish extracts intended for multiresidue determination of pesticides by UHPLC-MS/MS. The material performance was investigated regarding the removal of matrix-interfering compounds, particularly phospholipids, and compared to sorbents conventionally used in d-SPE procedures. In addition, the analytical method was validated for 130 pesticides and applied to commercial fish fillet samples to evaluate the feasibility of the biosorbent as a low-cost, sustainable alternative.

## 2. Materials and Methods

### 2.1. Chemicals, Reagents, and Materials

The reagents, solvents, and other materials used in this study were: HPLC-grade acetonitrile (Biograde, Waltham, MA, USA); 100% glacial acetic acid, 98% formic acid, and HPLC-grade methanol (Sigma-Aldrich, Taufkirchen, Germany); analytical grade sodium chloride, analytical grade ammonium formate, analytical grade sodium hydrogen citrate sesquihydrate, and analytical grade anhydrous magnesium sulfate (Sigma-Aldrich, Burlington, MA, USA); Extran^®^ neutral (Merck, São Paulo, Brazil); ultrapure water from a Milli-Q Direct 3UV^®^ system with a resistivity of 18.2 MΩ·cm (Millipore, Molsheim, France).

The neat pesticide standards and the surrogate standard deuterated atrazine (atrazine-d_5_) were from LGC Standards (Augsburg, Germany) with purity > 98%. The stock solutions of each compound were prepared in acetonitrile at 1000 mg L^−1^, considering the purity of the standards. To check the performance of the chromatographic analysis, triphenylphosphate 99% purity (Sigma-Aldrich, Burlington, MA, USA) was selected as internal standard.

The following sorbents were evaluated during the cleaning step: chitosan-modified vegetable sponge prepared in the laboratory; chitosan from crab shells (Sigma-Aldrich, Tokyo, Japan); octadecylsilane (C18) (Sigma-Aldrich, Burlington, MA, USA); primary-secondary amine (PSA) (Supelco, Bellefonte, PA, USA); Captiva EMR-Lipid (Agilent Technologies, Santa Clara, CA, USA); Phree™ Phospholipid Removal, 1 g/1 mL (Phenomenex, Torrance, CA, USA). We also used 13-mm nylon filters with a pore size of 0.22 μm (Allcrom, São Paulo, Brazil); 2- and 4-mL glass vials (Agilent Technologies, Santa Clara, CA, USA), 50- and 15-mL polypropylene (PP) Falcon tubes (Sarstedt, Sarstedt, Germany), Argon 5.0 (Air Liquide, São Paulo, Brazil), and standard laboratory glassware.

### 2.2. Instrumentation

Pesticide analysis was performed using a Xevo TQ UHPLC-MS/MS system (Waters, Milford, MA, USA) equipped with an Acquity binary pump and an autosampler. For the separation of the 130 pesticides, an Acquity UPLC™ BEH C18 column (50 × 2.1 mm i.d.; 1.7 μm; Waters, Milford, MA, USA) was used, with an injection volume of 10 µL and a column temperature of 40 °C. The mobile phase consisted of (A) water:methanol (98:2, *v*/*v*) and (B) methanol:water (98:2, *v*/*v*), both containing 5 mmol L^−1^ of ammonium formate and 0.1% (*v*/*v*) formic acid. Elution was performed in gradient mode as follows: 0 to 0.25 min, 5% B; increasing to 100% B by 7.75 min, held until 8.50 min; and 8.51 to 10.00 min, 5% B. The mobile phase flow rate was 0.225 mL min^−1^, resulting in a total chromatographic run time of 10 min. Nitrogen was used as the desolvation gas at a flow rate of 600 L h^−1^, while the collision gas was maintained at 0.15 mL min^−1^. The mass spectrometer operated with electrospray ionization in positive mode (ESI+). Data acquisition was performed in selected reaction monitoring (SRM) mode, with two transitions monitored for each analyte. The most abundant transition was used for quantification, while the second highest-intensity transition was used to confirm the identification of the analyzed compounds (Table S1).

Microstructural analysis of the biosorbent was performed by scanning electron microscopy (SEM) using a JEOL JSM-6360 microscope (Tokyo, Japan), equipped with a QUEST energy-dispersive X-ray spectroscopy (EDS) system from ThermoSystem (Hillsboro, OR, USA). Fourier transform infrared (FTIR) spectra were obtained using a Shimadzu IRPrestige-21 spectrometer (Kyoto, Japan). Both analyses were performed in laboratories at the Federal University of Santa Maria (UFSM).

### 2.3. Preparation of the Chitosan-Modified Vegetable Sponge Sorbent

To prepare the vegetable sponge-based sorbents, the sponge was first cut and ground. Next, 1 g of the ground sponge was immersed in a 1 mol L^−1^ acetic acid solution containing 1 g of chitosan and left in contact at room temperature for 12 h. After this period, the material was washed three times with distilled water to remove excess reagents and then dried in an oven at 80 °C until a constant mass was reached. The resulting sorbent was characterized and used in subsequent steps.

### 2.4. Sampling and Sample Preparation

The 20 commercial fish fillet samples used in this study were purchased at supermarkets in the central region of the state of Rio Grande do Sul, Brazil. After sampling, the fillet tissue was separated, homogenized using a mixer, and separated for sample preparation.

The extraction method was adapted from Munaretto et al. [33], with modifications to the cleaning step. Initially, approximately 5 g of sample were weighed and transferred to a 50-mL Falcon tube. Next, 10 mL of acetonitrile containing 0.1% (*v*/*v*) formic acid were added, and the tube was vortexed for 1 min to facilitate the extraction of the analytes. Next, 4 g of anhydrous magnesium sulfate (MgSO_4_) and 1 g of sodium chloride (NaCl) were added, and the tube was vortexed for 1 min. The tube was then centrifuged at 4850× *g* for 8 min at 4 °C, and 1 mL of the supernatant was transferred to a 15-mL Falcon tube for the extract clean-up step.

To evaluate the extract clean-up step, different strategies were compared; their experimental conditions are presented in Table 1. The extract from the fortified blank sample at a concentration of 0.005 mg kg^−1^ was used. In tests T1 and T2, the SPE pass-through mode was employed, using 1 mL of the extract in Captiva EMR-Lipid^®^ and Phree™ cartridges, designed primarily for the removal of lipids and phospholipids, respectively. In the d-SPE tests, 25 mg of PSA and 125 mg of C18 (T3), sorbents that work synergistically to remove polar and nonpolar interferents, or 100 mg of chitosan-modified vegetable sponge (CMVS) (T4) were added to 1 mL of the extract. Additionally, the emulsification/demulsification procedure based on the work described by Peng et al. [34] was evaluated using 2 mL of the extract, without the addition of a sorbent (T5). The tube was centrifuged at 4650× *g* for 6 min before analysis.

### 2.5. Method Validation

Method validation was performed using salmon fillet samples free of the analytes of interest. Validation was evaluated as recommended by SANTE guideline [35]. Recovery and precision studies were conducted in repeatability (R), in the same day, and intermediate precision (IP), on three consecutive days, assay using seven replicates at the fortification levels 0.001, 0.005, 0.010, and 0.020 mg kg^−1^. Recoveries were calculated as the ratio of the peak area obtained for the fortified sample to the peak area of the matrix-matched standard at the same concentration. Linearity was evaluated using analytical curves prepared in matrix at concentrations of 0.5, 1.0, 2.5, 10, 20, and 50 μg L^−1^, with the aim of compensating for possible MEs effects and ensuring the reliability of the quantification. The ME was determined by comparing the slopes of the analytical curves prepared in acetonitrile and in the blank extract of the matrix as described by Nieto-Garcia et al. [36].

## 3. Results and Discussion

### 3.1. Effect of Dilution Conditions on the Determination of Pesticide Residues by UHPLC-MS/MS

The selected instrumental conditions allowed for the simultaneous monitoring of 130 pesticides, using two transitions for each compound in SRM monitoring mode, in a 10-min chromatographic run. During the evaluation of the final composition of the extracts, it was observed that dilution in the mobile phase resulted in broader peaks with greater tailing, while dilution in water produced more defined and symmetrical peaks. Thus, a twofold dilution in ultrapure water was selected to avoid a high dilution factor and maintain adequate sensitivity.

### 3.2. Characterization of the Biosorbent

The activation of the chitosan-modified vegetable sponge results in a three-dimensional fibrous structure composed mainly of cellulose, hemicellulose, and lignin. In this context, acetic acid was used as both an activating agent and a solvent for dissolving the chitosan. According to Wen et al. [37], acid treatment induces modifications on the cellulose surface through the partial hydrolysis of glycosidic bonds, resulting in the exposure of additional hydroxyl groups. These groups can act as interaction sites for the immobilization of chitosan, promoting the formation of hybrid material with greater availability of functional groups for sorting processes.

**Figure 1:**
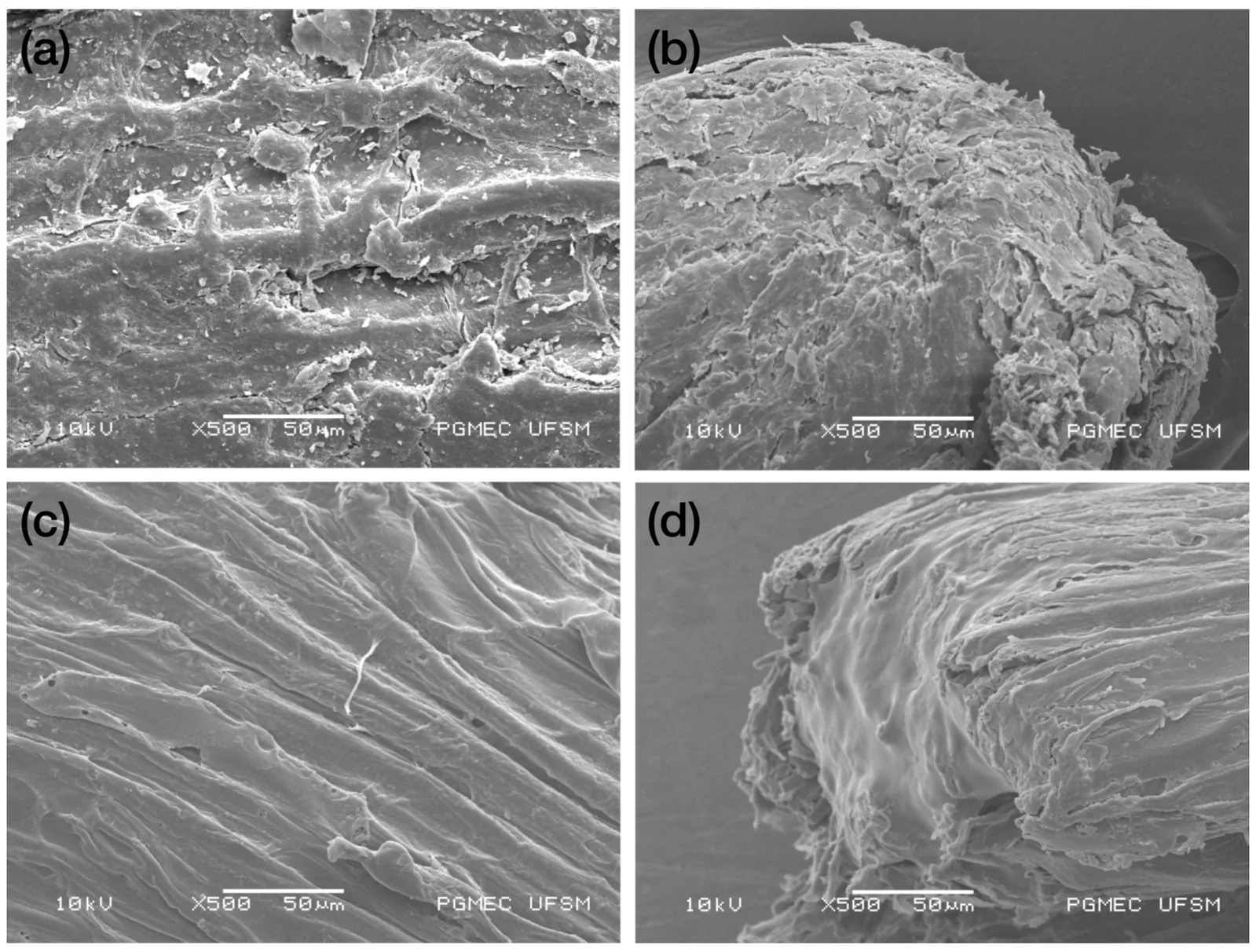
Scanning electron microscopy (SEM) images of (**a**,**b**) the unmodified vegetable sponge, exhibiting a rough surface and highly porous three-dimensional structure, and (**c**,**d**) the chitosan-modified vegetable sponge, showing a smoother surface. Surface morphology at 500× magnification (**a**,**c**) and cross-sectional morphology at 500× magnification (**b**,**d**).

The morphological characteristics of the natural sponge and the chitosan-modified sponge were evaluated by scanning electron microscopy (SEM). As shown in Figure 1a,b, the unmodified sponge exhibited a rough surface associated with a highly porous three-dimensional structure. After modification with chitosan (Figure 1c,d), a reduction in surface roughness was observed due to the coating of the fibers by the chitosan polymer layer. Despite this change, the three-dimensional porous structure was preserved, indicating that the modification process did not compromise the material architecture. The preservation of this porous network is particularly important, as it promotes the diffusion of analytes and the availability of adsorption sites during the extraction process.

**Figure 2:**
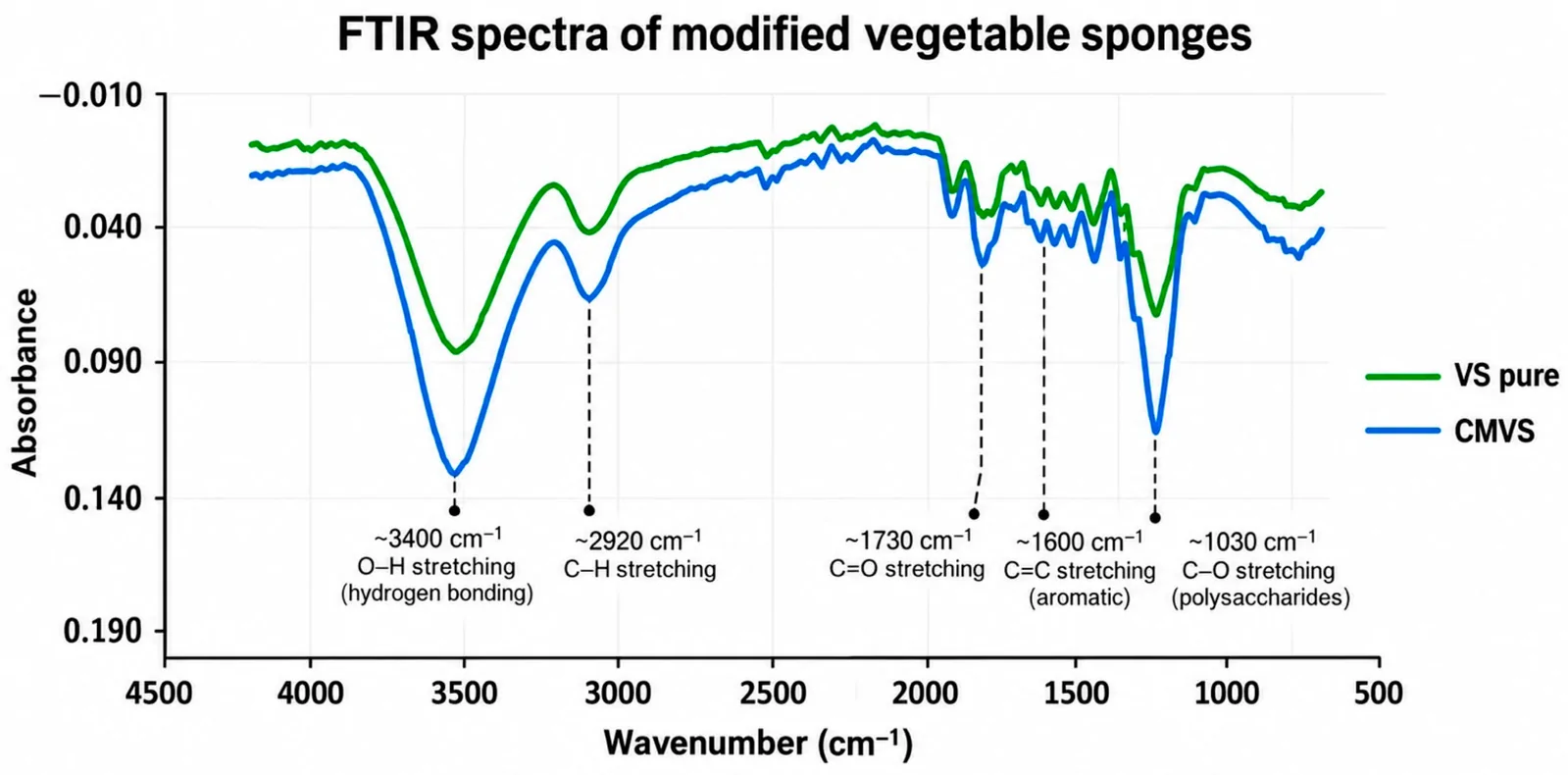
FTIR spectra of pure vegetable sponge (VS pure) and chitosan modified vegetable sponge (CMVS). The spectra were recorded in the range of 4000–500 cm^−1^. The main absorption bands are assigned to O–H stretching (~3400 cm^−1^), C–H stretching (~2920 cm^−1^), and C–O stretching (~1030 cm^−1^). Differences in band intensities between VS pure and CMVS indicate chemical changes resulting from the surface modification of the vegetable sponge.

The FTIR spectra of the unmodified sponge and the chitosan-modified sponge are shown in Figure 2. Spectroscopic analysis was performed to identify the functional groups present and to verify the structural changes resulting from the modification process.

In the FTIR spectrum of the modified sponge, changes were observed in the regions of 3500–3300 cm^−1^, 1600–1550 cm^−1^, and 1150–1000 cm^−1^, which can be attributed to the presence of functional groups characteristic of chitosan, such as hydroxyl (–OH), amine (–NH_2_), and amide. These spectral changes suggest the occurrence of interactions between chitosan and the s that make up the sponge cell wall, primarily cellulose and hemicellulose. Furthermore, the results provide evidence of chitosan incorporation into the plant matrix, possibly through hydrogen bonding and polymer chain rearrangements, consistent with observations previously reported by Zhou et al. [38].

### 3.3. Clean-Up Performance

The performance of the different clean-up strategies was evaluated based on the number of compounds that exhibited recoveries between 70 and 120%, with a relative standard deviation (RSD) ≤ 20%, using a blank sample fortified at 0.005 mg kg^−1^. The results are presented in Table 2.

Among the procedures evaluated, the Phree™ sorbent performed best, yielding acceptable recoveries for 134 compounds, followed by the chitosan-modified vegetable sponge (CMVS), which yielded satisfactory results for 130 compounds. The four compounds that the commercial sorbent Phree™ recovered but that the biosorbent CMVS did not recover were fenazaquine, flusilazole, furatiocarb, and hexitiazox. The PSA+C18 system recovered 124 compounds, while emulsification/demulsification and EMR-Lipid^®^ performed less well, with 116 and 115 compounds meeting the SANTE [35]. acceptance criteria, respectively. Although it did not outperform Phree™, the modified sponge yielded results comparable to those of commercial sorbents widely used in QuEChERS methods, with recoveries ranging from 71 to 118%. These results suggest that modifying the vegetable sponge with chitosan provided active sites capable of promoting the removal of matrix interferents without significantly compromising the recovery of analytes. Furthermore, the preservation of the sponge porous structure after functionalization may have contributed to efficient cleaning of the extract, maintaining high surface availability for interaction with and removal of co-extractives. The emulsification/demulsification step yielded acceptable recoveries for 116 compounds. However, the evaluation by UHPLC-MS/MS of phospholipid removal (Figure S1) indicated lower efficiency compared to the modified sponge, suggesting that its use alone may not be sufficient for matrices with high lipid content. The results obtained demonstrate that the modified sponge constitutes a promising alternative to conventional commercial sorbents for the clean-up of fish fillet extracts. In addition to exhibiting superior analytical performance compared to PSA+C18 and EMR-Lipid^®^ sorbents, the biosorbent offers advantages associated with the use of renewable and low-cost materials, reinforcing its potential for application in multiresidue methods aimed at determining pesticides in complex matrices.

The results presented in Figure S1 corroborate the modified sponge performance in removing lipid compounds from the matrix. In conjunction with the recovery studies, it was observed that this material was capable of efficiently cleaning the extract without significantly compromising the recovery of the analytes. This behavior may be related to the presence of amino and hydroxyl groups in the chitosan structure, which favor different types of interactions with matrix components, including hydrogen bonding and electrostatic interactions. These characteristics may contribute to the retention of lipids and phospholipids, while simultaneously preserving analytes with distinct physicochemical properties [38].

### 3.4. Sustainability Assessment of the Developed Method

The incorporation of the principles of green analytical chemistry has encouraged the development of tools capable of measuring the environmental impact of analytical procedures. In this study, two complementary metrics were used: AGREEprep (Analytical Greenness Metric for Sample Preparation) version 0.91 and BAGI (Blue Applicability Grade Index) version 0.9. AGREEprep was used to assess the sustainability of the sample preparation step, considering ten criteria related, among other aspects, to the consumption of solvents, reagents, and materials, sample quantity, waste generation, energy consumption, and procedure productivity [39].

BAGI, in turn, was used to assess the practicality and operational applicability of the analytical method. This tool considers ten attributes, including the type of analysis, the number of analytes determined simultaneously, the required instrumentation, the number of samples prepared simultaneously, the sample preparation procedure, analytical productivity, the reagents and materials used, the need for preconcentration, the degree of automation, and the sample volume used [40]. Thus, the combined use of these two metrics made it possible to evaluate both the environmental performance of the sample preparation step and the practical feasibility of the method in routine analyses.

**Figure 3:**
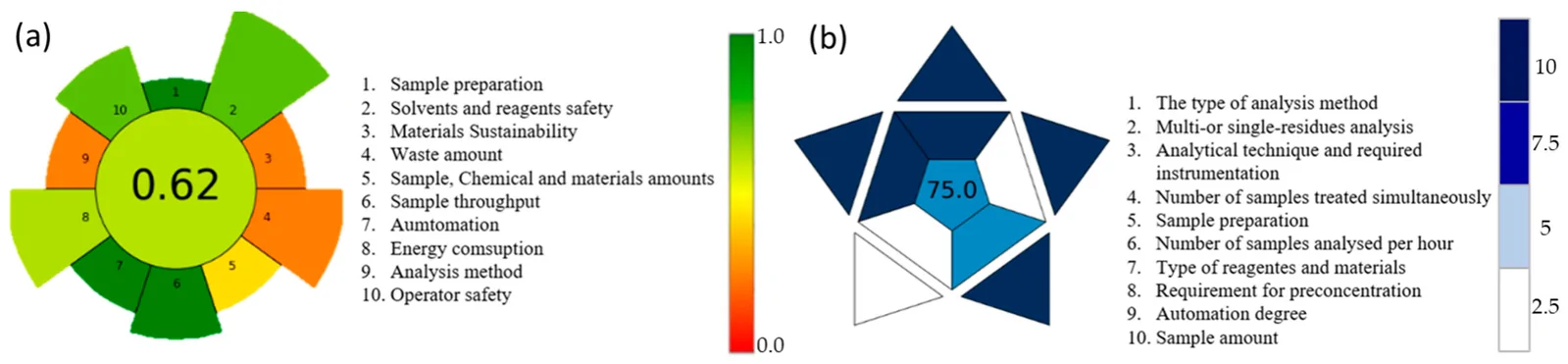
Evaluation of the sustainability of the developed method using the (**a**) AGREEprep and (**b**) Blue Applicability Grade Index (BAGI) metrics. The color scales represent the performance of each criterion, with higher scores indicating greater sustainability and applicability.

As shown in Figure 3a, the developed method achieved a sustainability score of 0.62 based on the AGREEprep metric. This result demonstrates satisfactory environmental performance, although there are still opportunities for improvement, particularly regarding sample preparation and environmental safety. Regarding the assessment using the BAGI index (Figure 3b), a more positive classification was observed with an overall score of 75.0, indicating that the procedure has good practical applicability from the perspective of Blue Analytical Chemistry. However, some parameters associated with analytical efficiency can still be improved to enhance the method overall effectiveness.

### 3.5. Method Validation Results

Table S2 presents the results of the method validation. A salmon fillet without the analytes under evaluation was selected for method validation. The matrix effect was evaluated by comparing the analytical curves prepared in solvent and in matrix extract, calculating the percentage variation between the slopes of the curves. Positive values indicate an increase in the analytical response, while negative values represent signal suppression. The matrix effect was classified as low when the absolute value was less than or equal to 20%, moderate when it ranged from 20 to 50%, and strong when it exceeded 50% [41]. This classification is frequently used in multiresidue methods to assess the intensity of interference from co-extracted components on the ionization of analytes. As examples, carbendazim exhibited a low matrix effect, with a 5.3% increase in signal, while atrazine exhibited a moderate effect, corresponding to 46.8%. For diazinon, a strong matrix effect was observed, with a 72.9% increase in the analytical response. These results demonstrate that the intensity of the matrix effect varies among compounds, even when analyzed in the same extract, justifying the use of calibration curves prepared in the matrix extract to compensate for changes in the instrumental response.

The evaluation of the matrix effect revealed different behaviors among the analyzed compounds. Carbendazim exhibited low suppression/enhancement of the analytical signal, with a matrix effect of 5.3%, indicating little influence of matrix components on the instrumental response. In contrast, atrazine exhibited a matrix effect of 46.8%, characterized as moderate, evidencing more significant interference from the matrix. Diazinon exhibited the highest observed matrix effect (72.9%), indicating a strong influence of the sample constituents on the analytical response. These results reinforce the importance of using matrix-matched calibration, as this strategy minimizes potential quantification errors resulting from matrix interference. The calibration curves were constructed using the matrix matched calibration covering a concentration range of 0.5 to 20.0 µg L^−1^, with correlation coefficients greater than 0.99, demonstrating adequate linearity of the method.

The accuracy and precision of the method were evaluated through recovery tests and determination of the relative standard deviation (RSD), respectively, using seven replicates of salmon fillet samples fortified at four concentration levels (0.001, 0.005, 0.010, and 0.020 mg kg^−1^). The intraday recovery values obtained for the validated levels ranged from 71 to 115%, remaining within the acceptance limits recommended by SANTE guidelines [35]. These results indicate adequate efficiency of the extraction procedure and good analytical performance, demonstrating that the method performs satisfactorily for the determination and monitoring of pesticide residues in fish samples. The method limits of quantification (LOQ) obtained considering the lower spike level that presented adequate recovery and precision, were 0.001 mg kg^−1^ for 109 compounds, 0.005 mg kg^−1^ for 21 compounds. The corresponding limits of detection (LOD) of 0.0003 and 0.0015 mg kg^−1^, respectively, indicating the method ability to detect and quantify pesticides at low concentrations. These limits are in general below the limits presented by similar works. Park et al. [42] obtained LOQ of 0.010 mg kg^−1^ for most of the 263 pesticides in flat fish analyzed by both GC-MS/MS and LC-MS/MS after a QuEChERS-based sample preparation. Donald et al. [43] analyzed salmon fillet by LC-QTOF MS with detection limits of 0.010 mg kg^−1^ or higher for presence/absence screen of 215 analytes. Collazo et al. [44] developed a QuEChERS-based method for the determination of 67 pesticide residues in fish muscle tissue, using gas and liquid chromatography coupled with mass spectrometry. Lee et al. [45] developed a QuEChERS and GC-MS/MS based method for the determination of 57 pesticide residues in fishery products, including flatfish. The LOQ values ranged from 0.003 to 0.007 mg kg^−1^. Kim et al. [46] developed a QuEChERS method coupled with LC-HRMS for the determination of 32 pesticides and 20 pharmaceuticals in aquaculture products. The authors obtained LOQ values between 0.0005 and 0.005 mg kg^−1^. The LOQ values obtained in the present study are in a similar range, with most compounds having an LOQ of 0.001 mg kg^−1^.

Table S3 [16,33,37,42,44,45,46,47,48,49,50,51,52,53,54,55,56,57,58,59] presents a comparison between the proposed method and the analytical methods previously described for the determination of pesticide residues in fish samples. The analytical performance of the proposed method, in terms of ease and cost of analysis and the achieved detectability, is similar to or even superior to that of the published methods.

### 3.6. Application of the Method to Fish Fillet Samples

Analysis of fish fillet samples revealed the presence of residues from various pesticides, as shown in Table 3, indicating contamination by compounds from different chemical classes. Among the identified analytes, atrazine had the highest concentration, quantified at 0.1308 mg kg^−1^ in sample A11. In addition to this compound, trifloxystrobin, fenpropimorph, epoxiconazole, propiconazole, pyraclostrobin, and clomazone were detected in concentrations ranging from 0.0010 to 0.0897 mg kg^−1^. For all identified compounds, the concentration values found were above the limit of quantification (LOQ = 0.001 mg kg^−1^) established for the method. These results confirm the ability of the developed analytical procedure to detect and quantify pesticide residues at trace levels in complex matrix samples, such as fish fillet tissue.

The presence of the detected compounds was confirmed by analysis of the UHPLC-MS/MS chromatograms, as illustrated in Figure S2. The chromatographic peaks corresponding to fenpropimorph, atrazine, and propiconazole demonstrate the unequivocal identification of these analytes in the evaluated samples. Of note is sample A11, in which the highest concentrations of both the herbicide atrazine (0.1308 mg kg^−1^) and of the fungicide propiconazole (0.0295 mg kg^−1^) were observed simultaneously, while sample A4 showed quantification of the fungicide fenpropimorph (0.0062 mg kg^−1^). All the pesticides found in the fish samples are currently used on a large scale in various crops.

The detection of pesticide residues in aquatic organisms is an important indicator of changes in environmental quality, since these compounds can enter aquatic environments through surface runoff, atmospheric transport, or leaching processes. Furthermore, due to the physicochemical characteristics of some pesticides, these compounds can persist in the environment and undergo bioaccumulation and transfer throughout the food chain, potentially affecting different trophic levels and posing potential risks to human health [60].

Recent studies demonstrate that the exposure of aquatic organisms to pesticides can trigger biochemical, physiological, and genetic changes, compromising processes such as fish growth, development, and reproduction [61]. Among the compounds identified in this study, atrazine deserves special attention due to its widespread agricultural use and the possible adverse effects associated with exposure, including endocrine and metabolic changes, as well as potential risks to human health [62]. Thus, the results obtained reinforce the importance of continuous monitoring of pesticide residues in aquatic environments and highlight the relevance of sensitive and reliable analytical methods for assessing contamination in animal-derived matrices.

## 4. Conclusions

This study enabled the development and application of an efficient analytical method for the multiresidue determination of pesticides in fish fillet samples at very low levels. The proposed procedure employed an extraction-based sample preparation step followed by a clean-up step using a biosorbent derived from a vegetable sponge modified with chitosan, which exhibited a high capacity for removing lipid interferents, thereby contributing to the reduction of the matrix effect and the production of extracts with higher analytical quality. The developed method demonstrated adequate selectivity, linearity, precision, and accuracy, and was validated for the determination of 130 compounds in fish. The low limits of quantification obtained demonstrate the sensitivity of the procedure and its suitability for determining pesticide residues at trace levels in a complex matrix, such as fish fillet tissue. Furthermore, the method exhibited satisfactory chromatographic performance, allowing for the reliable separation, identification, and quantification of the analytes evaluated.

The application of the method to real samples revealed the presence of residues from various pesticides in some of the fish samples analyzed, demonstrating the occurrence of these contaminants in aquatic environments. These results underscore the importance of continuous monitoring of pesticide residues in aquatic organisms, given the potential environmental impacts and risks associated with human exposure. Thus, the developed method has potential for application in food safety assessments, contributing to the advancement of more efficient and sustainable analytical strategies for the analysis of contaminants in complex matrices.

## Figures and Tables

**Table 1:** Experimental conditions evaluated during the clean-up stage of fish fillet extracts.

Test	Clean-Up		Sorbent or Procedure
	Pass-Through SPE	d-SPE	
T1	1 mL	-	EMR-Lipid^®^
T2	1 mL	-	Phree™
T3	-	1 mL	25 mg PSA + 125 mg C_18_
T4		1 mL	100 mg CMVS
T5		2 mL	Emulsification/demulsification

**Table 2:** Comparison of different clean-up sorbents based on the number of compounds showing acceptable recoveries (70–120%) and RSD (≤20%).

Sorbent or Procedure	Number of Compounds with Acceptable Results
EMR-Lipid^®^	115
Phree™	134
PSA + C18	124
CMVS	130
Emulsification/Demulsification	116

**Table 3:** Pesticide residues in fish samples and their respective concentrations (mg kg^−1^).

Sample	Compound	Concentration (mg kg^−1^)
A2	Trifloxystrobin	0.0024
A4	Fenpropimorph	0.0062
A11	Atrazine	0.1308
	Epoxiconazole	0.0247
	Propiconazole	0.0295
	Pyraclostrobin	0.0344
	Trifloxystrobin	0.0897
A16	Atrazine	0.0010
A18	Clomazone	0.0031
	Fenpropimorph	0.0031

## Data Availability

The original contributions presented in the study are included in the article and Supplementary Material. Further inquiries can be directed to the corresponding author.

## Supplementary Materials

The following supporting information can be downloaded at: https://www.mdpi.com/article/10.3390/foods15203696/s1, Figure S1: Representative UHPLC-MS/MS chromatograms for phospholipids in fish sample extracts exhibiting different levels of matrix interference. Panels A–F compare high- and low-interference matrices based on the signal intensity of co-extracted compounds. Different colors indicate the MS transition monitored; Figure S2: Representative chromatograms of positive fish fillet samples containing fenpropimorph, atrazine, and propiconazole and the signal for the respective limit of quantification (LOQ); Table S1: Compounds determined by UHPLC-MS/MS, with their respective retention times (t_R_), cone voltage (CV), precursor and product ions, and respective collision energies (CE); Table S2: Validation results for the QuEChERS method: LOQ, LOD, linearity (r^2^), accuracy, as recovery, precision, as RSD, in terms of repeatability (R) and intermediate precision (IP) and matrix effect; Table S3: Comparison of sample preparation and analysis methods for pesticide residues in fish fillets with their respective limits of quantification (LOQ).
