# Supplementary material for: Evaluation of a Luffa Cylindrica-Based Biosorbent as an Alternative for Sample Preparation in the Multiresidue Determination of Pesticides in Fish Fillets by UHPLC-MS/MS
Source: Foods. 2026 Oct 17;15(20):3696. doi: 10.3390/foods15203696 (PMC13704407; doi:10.3390/foods15203696)
Supplement: Supplementary file 1 [file foods-15-03696-s001.zip › foods-4574751-supplementary.pdf]

Article

Evaluation of a Luffa cylindrica-Based Biosorbent as an Alternative for Sample Preparation in the Multiresidue Determination of Pesticides in Fish Fillets by UHPLC-MS/MS

Franciele Fátima Machado [1], José Dilson Francisco da Silva [2], Luana Floriano [1], Juliana Diniz de Barros Kuntz [1], Alcides Ferreira Coradini [1], Osmar Damian Prestes [1] and Renato Zanella [1,*]

1 Chemistry Department, Federal University of Santa Maria (UFSM), Santa Maria 97105-900, RS, Brazil; francimachadote@gmail.com (F.F.M.); luanaflorianoqmc@gmail.com (L.F.); juukuntz30@gmail.com (J.D.B.K.); alcidesfcoradini@gmail.com (A.F.C.); osmar.prestes@ufsm.br (O.D.P.)

2 Federal Institute of Education, Science, and Technology of Tocantins, Araguaina 77824-838, TO, Brazil; dilson13@gmail.com (J.D.F.d.S.)

* Correspondence: renato.zanella@ufsm.br; Tel.: +5555 3220-8011

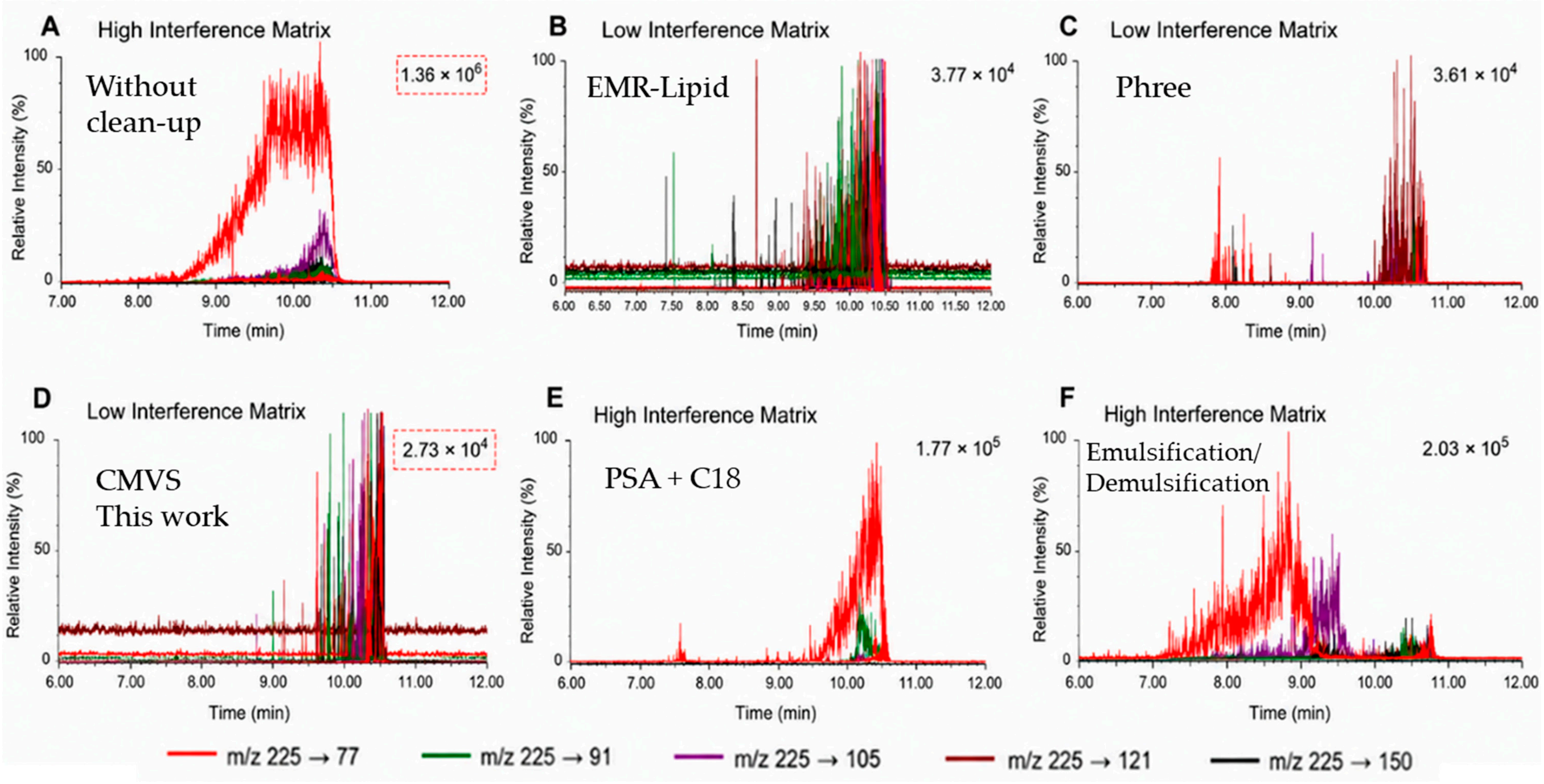

Figure S1 - Representative UHPLC-MS/MS chromatograms for phospholipids in fish sample extracts exhibiting different levels of matrix interference. Panels A–F compare high- and low-interference matrices based on the signal intensity of co-extracted compounds. Different colors indicate the MS transition monitored.

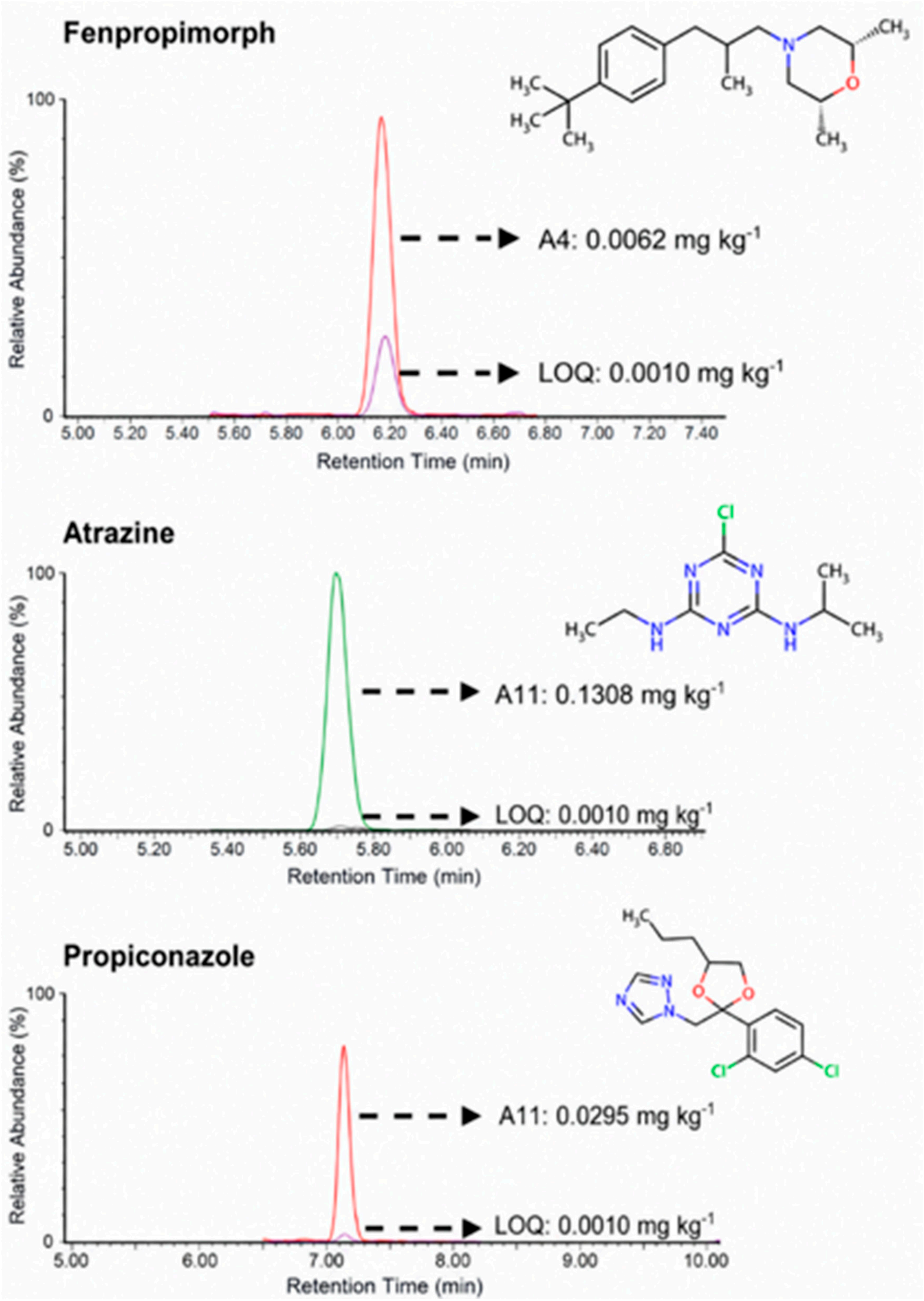

Figure S2 - Representative chromatograms of positive fish fillet samples containing fenpropimorph, atrazine, and propiconazole and the signal for the respective limit of quantification (LOQ).

Table S1. Compounds determined by UHPLC-MS/MS, with their respective retention times (t_R), cone voltage (CV), precursor and product ions, and respective collision energies (CE).

Nr.	Compounds	t_R (min)	CV (V)	Precursor ion (*m*/*z*)	1st transition Quantification		2nd transition Identification	
					Product ion (m/z)	CE (eV)	Product ion (m/z)	CE (eV)
1	Acetamiprid	3.69	23	223.0	56.1	15	126.0	20
2	Ametryn	5.65	32	228.1	68.1	36	186.1	18
3	Atrazine	5.51	30	216.1	96.1	23	174.1	18
4	Azaconazole	5.91	34	300	159.1	28	231.1	18
5	Azamethiphos	4.93	31	325	111.9	35	138.9	24
6	Azoxystrobin	5.97	17	404.0	329.0	30	372.0	15
7	Bitertanol	7.10	12	338.1	70.1	8	99.1	16
8	Boscalid	6.18	32	342.9	139.9	20	307.0	20
9	Bromuconazole	6.40	32	376.0	70.1	25	158.9	35
10	Buprofezin	7.51	22	306.1	57.4	20	201.0	12
11	Piperonyl butoxide	7.75	20	356.3	176.9	10	119	35
12	Carbaryl	5.14	19	202.0	117.0	28	145.0	22
13	Carbendazim	2.76	24	192.1	132.1	28	160.1	18
14	Carbofuran	4.94	25	222.1	123.0	16	165.1	16
15	Carboxin	5.08	25	236.0	87.0	22	143.0	16
16	Cyanazine	5.25	28	241.0	96.0	25	241.0	17
17	Cymoxanil	3.91	14	199.0	111.0	18	128.0	8
18	Cyazofamid	6.79	25	325	107.9	15	261	10
19	Cyproconazole	6.68	5	292.2	70.2	20	125.1	30
20	Clofentezine	7.26	20	303.0	102.0	35	138.0	15
21	Chlorantraniliprole	5.82	20	481.6	451.1	22	283.9	14
22	Chlorfenvinphos	7.14	28	358.9	155.0	12	99.0	30
23	Chlorimuron-ethyl	6.22	25	415.1	213.0	17	186.0	15
24	Chlorpyrifos-methyl	7.24	23	321.8	125.0	20	289.9	16
25	Clothianidin	3.59	25	250	169	10	132	15
26	Clomazone	5.90	23	240.0	89.0	46	125.0	18
27	Demeton-S-methyl sulfone	2.98	32	263	169.1	17	121	17
28	Diclosulam	5.45	46	406.1	161	30	378	15
29	Dichlorvos	5.05	23	221.1	109.0	22	79.0	34
30	Dicrotophos	3.38	30	238	112	10	193	10
31	Diazinon	6.96	20	305.1	96.9	35	169.0	22
32	Diflubenzuron	6.91	34	311.1	158.1	12	141.1	28
33	Difenoconazole	7.21	37	406.0	111.1	60	251.1	25
34	Dimethoate	3.63	12	230.1	199.0	10	125.0	20
35	Dimoxystrobin	6.99	20	327.1	205.2	10	116.1	21
36	Diniconazole	7.40	10	326.1	70.2	25	326.1	159
37	Diuron	5.69	27	233.0	46.3	14	72.1	18
38	EPN	7.42	22	324	157	25	296	14
39	Epoxiconazole	6.58	25	330.0	101.0	50	121.0	22
40	Ethiofencarb	5.47	10	226.1	107	15	164	10
41	Ethoprophos	6.77	18	242.9	130.9	20	97	31
42	Ethyl azynphos	6.50	10	346.0	77.1	36	132.0	16
43	Ethyl pirimiphos	7.71	42	334.1	198.1	23	182.1	25
44	Famoxadone	6.98	12	392.2	238.0	20	331.1	10
45	Fenamidone	6.15	22	312.1	236.1	14	92.0	25
46	Fenamiphos	6.72	27	304.1	202.1	36	217.1	24
47	Fenarimol	6.56	17	331.0	81.0	34	268.0	22
48	Fenhexamid	6.70	35	301.9	97.1	25	55.1	35
49	Fenoxycarb	6.95	10	302.1	88	20	116.1	11

Nr.	Compounds	t_R (min)	CV (V)	Precursor ion (*m/z*)	1st transition Quantification		2nd transition Identification	
					Product ion (m/z)	CE (eV)	Product ion (m/z)	CE (eV)
50	Fenpyroximate	7.89	23	422.2	138.1	32	336.1	15
51	Fenpropimorph	6.00	41	304.2	147.1	28	57.2	30
52	Fenthion	6.90	25	279.1	169.1	16	247.1	13
53	Fluazifop-P-butyl	7.62	38	384.1	282.1	22	328.1	16
54	Flutriafol	5.56	23	302.1	123.1	29	70.2	18
55	Furathiocarb	7.63	20	383.3	194.9	15	252	10
56	Hexythiazox	7.72	21	353.0	168.1	26	228.1	14
57	Imazalil	5.45	31	297.0	69.0	22	159.0	22
58	Imazamox	4.12	25	306.1	278	20	218	30
59	Imazapyr	3.53	38	262.2	69.2	26	86.1	26
60	Imazaquin	5.07	40	312.2	267.2	20	86.2	28
61	Imazethapyr	4.75	45	290.1	245.2	20	177.1	25
62	Imidacloprid	3.33	23	256.1	175.1	20	209.1	15
63	Iprovalicarb	6.46	19	321.1	203.1	10	119.1	16
64	Kresoxim-methyl	6.83	15	314.1	116.0	12	206.0	7
65	Linuron	6.07	25	249.1	181.1	16	160.1	18
66	Malathion	6.27	12	331.0	99.0	24	127.0	12
67	Mecarbam	6.54	12	330.0	97.0	35	227.1	8
68	Mephosfolan	5.00	34	270.1	140	24	75.1	22
69	Mepronil	6.29	27	270.1	91.0	44	119.0	28
70	Metalaxyl	5.62	15	280.1	192.1	17	220.1	13
71	Metconazole	7.08	29	320.1	125.0	36	70.0	22
72	Methyl azinphos	5.82	12	318.0	261.0	8	160.0	8
73	Methidathion	5.94	30	303	144.8	10	303	84.9
74	Methiocarb	6.15	19	226.0	121.0	22	169.0	10
75	Methiocarb sulfone	4.02	19	226.0	121.0	22	169.0	10
76	Methoxyfenozide	6.47	15	369.2	149.1	15	313.2	10
77	Metsulfuron-methyl	4.88	22	382.0	198.9	22	167.0	16
78	Metribuzin	5.02	5	215	131	20	89	20
79	Mevinphos	3.62	15	225.1	121.1	15	193.1	10
80	Methyl pirimiphos	7.43	42	306.3	164.1	22	108.1	30
81	Myclobutanil	5.00	25	289.1	70.2	15	125.1	30
82	Molinate	6.54	17	188	126	13	55	24
83	Monocrotophos	2.97	15	224.1	98.1	12	127.1	16
84	Monolinuron	5.24	23	215.0	99.0	34	126.0	22
85	Nicosulfuron	5.01	26	411	182	22	106	32
86	Nuarimol	6.27	25	315.0	252.0	20	81.1	15
87	Oxadixyl	4.73	20	279.1	219	12	132.3	25
88	Oxamyl	2.46	12	237.0	90.0	10	72.0	10
89	Ethyl paraoxon	6.00	28	276.1	220.1	17	248.2	16
90	Pencycuron	7.33	30	329.1	124.9	30	281	16
91	Penconazole	7.08	15	284	70.1	15	159	25
92	Penoxsulam	5.40	42	484.2	195.1	30	164.1	40
93	Picoxystrobin	6.90	10	368	145.1	25	205.1	10
94	Pyraclostrobin	7.26	25	388.1	163.0	25	193.9	12
95	Pyrazophos	7.35	33	374.0	222.1	22	194.0	32
96	Pyridaphenthion	6.46	31	341.0	189.0	22	92.0	34
97	Pyrimethanil	5.75	25	200.0	107.0	24	82.0	24
98	Phosalone	7.26	12	367.9	181.9	14	110.9	42
99	Phosmet	6.05	258	318	160	22	77	46
100	Phorate	7.27	15	261	75	12	199	5
101	Pirimicarb	4.29	25	239.1	72.4	18	182.1	15

Nr.	Compounds	t_R (min)	CV (V)	Precursor ion (*m/z*)	1st transition Quantification		2nd transition Identification	
					Product ion (m/z)	CE (eV)	Product ion (m/z)	CE (eV)
102	Pyriproxyfen	7.80	5	322.1	96.0	15	227.1	10
103	Procymidone	6.92	10	368.0	145.0	22	205.0	10
104	Propanil	6.32	34	217,9	127	22	161,9	16
105	Prometryn	6.36	25	242.0	158.0	25	200.1	20
106	Quinalphos	6.98	15	299.0	162.9	24	96.9	30
107	Quizalofop-P-ethyl	7.59	30	373.0	299.1	18	91.1	32
108	Saflufenacil	6.08	26	501.1	198.0	38	349.0	20
109	Simazine	5.07	40	202.0	124.0	16	96.0	22
110	Spinosad A	7.04	47	732.6	98.1	59	142.0	31
111	Spinosad D	7.26	42	746.5	98.1	53	142.0	31
112	Tebuconazole	7.11	30	308.2	70.1	24	124.9	40
113	Tebufenozide	6.92	10	353.2	133.1	10	105.1	20
114	Tebufenpyrad	7.68	15	334.0	145.0	25	117.0	25
115	Tetraconazole	6.78	15	372.0	159.0	25	70.1	20
116	Thiabendazole	3.30	45	202.0	174.9	25	130.9	30
117	Thiacloprid	4.24	35	253.0	125.8	20	90.0	40
118	Thiamethoxam	3.03	25	292.0	132.0	20	211.2	10
119	Thiobencarb	7.32	25	258.1	125.1	15	89.1	35
120	Thiophanate-methyl	5.03	22	343	151	22	93	46
121	Triazophos	6.61	31	314,1	161,9	18	118,9	35
122	Triadimefon	3.82	30	294.1	69.1	20	196.9	20
123	Triadimenol	6.68	30	296.1	70.0	10	98.9	15
124	Tricyclazole	4.35	10	190.0	163.0	20	136.0	25
125	Trichlorfon	3.82	66	256,9	109,1	25	127	8
126	Trifloxystrobin	7.44	25	409.2	185.9	14	145.0	40
127	Triflusulfuron	5.74	30	438.0	182.0	20	139.0	45
128	Triflumizole	7.52	15	346.1	278.0	10	73.1	18
129	Triflumuron	7.25	5	359.0	156.1	20	139.1	30
130	Vamidothion	3.84	25	288.0	146.0	10	188.0	25

Table S2. *V*alidation results for the QuEChERS method: LOQ, LOD, linearity (r^2), accuracy, as recovery, precision, as RSD, in terms of repeatability (R) and intermediate precision (IP) and matrix effect.

Nr.	Compounds	r^2	LOQ µg kg⁻¹	LOD µg kg⁻¹	Recovery (%) (Precision, RSD_R %) Spiking levels (µg kg⁻¹), n = 7				Recovery (%) (RSD_{IP} %) Spiking levels (µg kg⁻¹), n = 7				Matrix effect (%)
					1	5	10	20	1	5	10	20	
1	Acetamiprid	0.9996	1	0.3	88 (6)	89 (4)	93 (3)	96 (3)	90 (6)	90 (5)	91 (5)	97 (3)	0.28
2	Ametryn	0.9973	1	0.3	74 (9)	72 (8)	78 (8)	77 (7)	69 (7)	70 (4)	85 (5)	90 (3)	50.8
3	Atrazine	0.9984	1	0.3	82 (12)	85 (6)	86 (7)	87 (6)	82 (11)	84 (6)	89 (3)	95 (3)	46.8
4	Azaconazole	0.9999	1	0.3	86 (10)	90 (12)	88 (7)	87 (7)	101 (9)	91 (6)	97 (7)	99 (2)	-0.02
5	Azamethiphos	0.9993	1	0.3	89 (9)	90 (10)	90 (12)	90 (11)	101 (16)	95 (5)	93 (4)	94 (7)	9.6
6	Ethyl azinphos	0.9957	1	0.3	85 (17)	73 (23)	87 (10)	107 (10)	121 (19)	103 (18)	105 (18)	107 (13)	43.0
7	Methyl azinphos	0.9993	1	0.3	93 (24)	81 (20)	92 (8)	96 (5)	121 (11)	91 (18)	94 (16)	102 (9)	20.3
8	Azoxystrobin	0.9977	1	0.3	70 (5)	80 (8)	83 (3)	84 (5)	86 (19)	86 (12)	94 (7)	94 (6)	15.4
9	Bitertanol	0.9966	1	0.3	95 (18)	92 (18)	85 (14)	90 (11)	103 (19)	74 (15)	84 (15)	95 (7)	-35.3
10	Boscalid	0.9979	1	0.3	81 (19)	78 (21)	92 (13)	93 (8)	65 (34)	76 (18)	95 (9)	97 (5)	44.6
11	Bromuconazole	0.9983	5	1.5	48 (45)	71 (26)	91 (8)	92 (12)	62 (50)	65 (22)	85 (19)	97 (6)	63.7
12	Buprofezin	0.9953	5	1.5	57 (8)	71 (13)	81 (5)	81 (4)	66 (5)	70 (5)	88 (7)	95 (5)	152.7
13	Piperonyl butoxide	0.9954	5	1.5	68 (5)	80 (10)	81 (13)	80 (11)	54 (14)	72 (12)	99 (11)	118 (3)	172.2
14	Carbaryl	0.9997	1	0.3	96 (9)	97 (12)	99 (9)	96 (11)	108 (9)	99 (7)	98 (6)	96 (3)	29.9
15	Carbendazim	0.9999	1	0.3	84 (2)	80 (7)	76 (4)	74 (6)	70 (3)	66 (11)	73 (6)	77 (2)	5.3
16	Carbofuran	0.9992	1	0.3	101 (7)	109 (4)	115 (4)	116 (5)	97 (8)	100 (8)	107 (7)	111 (4)	3.9
17	Carboxin	0.9976	5	1.5	65 (6)	79 (9)	89 (7)	89 (8)	94 (6)	90 (3)	96 (3)	98 (3)	28.8
18	Cyanazine	0.9997	1	0.3	90 (10)	93 (5)	97 (12)	97 (9)	94 (12)	88 (10)	101 (5)	97 (7)	-16.2
19	Cymoxanil	0.9995	1	0.3	90 (7)	93 (10)	102 (6)	104 (5)	90 (5)	89 (5)	93 (5)	95 (5)	1.5
20	Cyazofamid	0.9950	1	0.3	84 (19)	78 (11)	88 (14)	90 (20)	71 (13)	73 (17)	98 (14)	109 (6)	31.5
21	Cyproconazole	0.9964	1	0.3	78 (20)	82 (12)	86 (13)	93 (11)	59 (29)	77 (14)	90 (10)	103 (4)	22.6
22	Clofentezine	0.9993	1	0.3	102 (20)	86 (20)	86 (19)	91 (16)	59 (73)	73 (10)	98 (18)	114 (8)	126.0
23	Chlorantraniliprole	0.9964	1	0.3	99 (11)	117 (21)	76 (14)	86 (15)	102 (30)	77 (20)	95 (19)	105 (13)	-9.9
24	Chlorfenvinphos	0.9978	1	0.3	74 (16)	84 (19)	99 (19)	98 (7)	84 (20)	83 (7)	94 (10)	106 (6)	67.3
25	Chlorimuron-ethyl	0.9983	5	1.5	66 (10)	82 (14)	87 (5)	95 (6)	90 (12)	103 (16)	98 (20)	98 (12)	-62.5
26	Chlorpyrifos-methyl	0.9986	1	0.3	84 (37)	87 (8)	87 (15)	93 (12)	92 (36)	77 (21)	98 (20)	98(12)	101.3
27	Clothianidin	0.9962	5	1.5	55 (5)	79 (10)	93 (11)	101 (8)	106 (8)	95 (8)	91 (10)	94 (5)	-0.37
28	Clomazone	0.9980	1	0.3	78 (9)	86 (8)	91 (5)	93 (4)	81 (5)	87 (5)	98 (5)	99 (1)	31.1
29	Kresoxim-methyl	0.9986	1	0.3	87 (76)	93 (40)	97 (10)	79 (16)	80 (25)	84 (9)	103 (25)	107 (23)	93.5
30	Demeton-S-methyl sulfone	0.9991	1	0.3	92 (6)	90 (5)	88 (5)	92 (6)	93 (16)	91 (8)	95 (5)	100 (3)	-19.1
31	Diclosulam	0.9980	1	0.3	73 (25)	86 (17)	95 (12)	97 (6)	76 (50)	81 (16)	100 (17)	97 (6)	-35.2

Nr.	Compounds	r²	LOQ µg kg⁻¹	LOD µg kg⁻¹	Recovery (%) (Precision, RSD_R %)				Recovery (%) (RSD_{IP} %)				Matrix effect (%)
					Spiking levels (µg kg⁻¹), n = 7				Spiking levels (µg kg⁻¹), n = 7				
					1	5	10	20	1	5	10	20	
32	Dichlorvos	0.9974	1	0.3	100 (19)	70 (23)	90 (10)	94 (10)	84 (17)	97 (15)	93 (9)	96 (5)	4.2
33	Dicrotophos	0.9997	1	0.3	89 (8)	88 (6)	87 (7)	86 (10)	105 (7)	93 (8)	93 (8)	93 (5)	-6.5
34	Diazinon	0.9980	1	0.3	79 (14)	85 (4)	93 (8)	93 (6)	77 (10)	84 (8)	94(4)	107 (4)	72.9
35	Diflubenzuron	0.9801	5	1.5	136 (13)	99 (29)	84 (17)	85 (14)	68 (34)	108 (24)	90 (22)	99 (19)	165.9
36	Difenoconazole	0.9966	1	0.3	83 (20)	80 (8)	89 (11)	94 (7)	75 (26)	86 (8)	94 (7)	98 (4)	32.5
37	Dimethoate	0.9989	1	0.3	82 (8)	92 (2)	97 (3)	98 (4)	84 (9)	90 (4)	96 (5)	97 (2)	12.0
38	Dimoxystrobin	0.9994	1	0.3	114 (17)	96 (16)	91 (12)	87 (11)	70 (20)	75(5)	90 (10)	105 (2)	85.3
39	Diniconazole	0.9956	1	0.3	96 (14)	81 (20)	104 (16)	96 (14)	88 (20)	87 (20)	105 (17)	96 (14)	31.7
40	Diuron	0.9993	1	0.3	82 (9)	85 (12)	83 (14)	85 (7)	72 (10)	84 (10)	94 (5)	97 (3)	13.8
41	EPN	0.9968	5	1.5	62 (30)	75 (18)	79 (12)	90 (15)	61 (29)	70 (17)	84 (13)	103 (6)	119.4
42	Epoxiconazole	0.9985	1	0.3	73 (9)	85 (6)	92 (6)	99 (4)	81 (16)	78 (6)	95 (6)	100 (6)	23.4
43	Spinosad A	0.9967	1	0.3	72 (11)	78 (9)	97 (13)	106 (5)	78 (11)	81 (9)	97 (10)	106 (4)	145.5
44	Spinosad D	0.9962	5	1.5	59 (40)	75 (11)	71 (12)	75 (7)	56 (58)	69 (45)	86 (20)	92 (19)	164.0
45	Ethiofencarb	0.9992	1	0.3	97 (8)	93 (10)	93 (8)	88 (10)	103 (5)	93 (10)	101 (7)	105 (5)	19.0
46	Ethoprophos	0.9966	1	0.3	70 (18)	82 (9)	89 (12)	97 (7)	70 (17)	72 (19)	97 (8)	104 (6)	59.7
47	Famoxadone	0.9942	1	0.3	107 (40)	100 (36)	101 (30)	82 (27)	76 (55)	85 (36)	100 (33)	106 (26)	33.1
48	Fenamidone	0.9976	1	0.3	78 (9)	82 (9)	93 (9)	93 (8)	81 (16)	90 (10)	98 (3)	105 (4)	-5.0
49	Fenamiphos	0.9947	1	0.3	120 (10)	95 (17)	77 (20)	70 (6)	80 (16)	92 (17)	94 (4)	99 (8)	74.0
50	Fenarimol	0.9928	1	0.3	89 (19)	70 (15)	95 (20)	97 (8)	97 (34)	83 (24)	88 (19)	96 (13)	21.8
51	Fenhexamid	0.9957	1	0.3	82 (15)	70 (18)	91 (12)	105 (9)	95 (19)	83 (18)	102 (8)	99 (5)	49.6
52	Fenoxycarb	0.9889	1	0.3	105 (14)	117 (19)	80 (10)	73 (15)	90 (35)	97 (23)	103 (16)	105 (11)	172.5
53	Fenpyroximate	0.9968	5	1.5	59 (15)	71 (8)	72 (18)	70 (13)	56 (14)	63 (20)	93 (6)	100 (3)	53.2
54	Fenpropimorph	0.9986	1	0.3	76 (6)	82 (2)	88 (4)	91 (4)	82 (9)	83 (4)	93 (6)	105 (5)	72.9
55	Fenthion	0.9909	5	1.5	56 (77)	65 (29)	83 (13)	89 (8)	81 (19)	79 (25)	114 (31)	102 (21)	77.3
56	Fluazifop-P-butyl	0.9951	1	0.3	79 (14)	82 (12)	91 (12)	89 (8)	56 (10)	63 (10)	91 (7)	106 (4)	119.1
57	Flutriafol	0.9893	1	0.3	106 (10)	113 (16)	82 (19)	78 (9)	92 (12)	74 (12)	86 (16)	102 (13)	-4.6
58	Phorate	0.9952	1	0.3	72 (14)	85 (9)	96 (8)	100 (5)	72 (13)	82 (6)	88 (10)	90(3)	71.3
59	Folpet	0.9981	1	0.3	86 (8)	86 (12)	90 (7)	89 (8)	71 (12)	89 (10)	97 (5)	101 (4)	96.8
60	Phosmet	0.9970	1	0.3	95 (18)	105 (15)	97 (10)	83 (11)	67 (47)	81 (20)	96 (13)	111 (7)	7.4
61	Furathiocarb	0.9962	1	0.3	89 (12)	84 (17)	92 (20)	99 (16)	66 (42)	83 (20)	84 (18)	118 (9)	49.9
62	Hexythiazox	0.9976	1	0.3	72 (20)	86 (10)	94 (11)	89 (10)	97 (13)	91 (9)	100 (9)	104 (5)	394.4
63	Imazalil	0.9949	1	0.3	68 (17)	84 (17)	84 (13)	82 (10)	37 (48)	65 (16)	81 (8)	85 (10)	50.8
64	Imazamox	0.9976	1	0.3	76 (16)	71 (12)	74 (14)	78 (12)	65 (19)	63 (9)	88 (7)	102 (5)	-12.8

Nr.	Compounds	r^2	LOQ µg kg⁻¹	LOD µg kg⁻¹	Recovery (%) (Precision, RSD_R %) Spiking levels (µg kg⁻¹), n = 7				Recovery (%) (RSD_{IP} %) Spiking levels (µg kg⁻¹), n = 7				Matrix effect (%)
					1	5	10	20	1	5	10	20	
65	Imazapyr	0.9984	1	0.3	77 (10)	87 (12)	83 (8)	85 (7)	70 (11)	71 (11)	89 (5)	112(5)	-20.1
66	Imazaquin	0.9999	1	0.3	71 (9)	64 (10)	71 (12)	73 (7)	55 (19)	54 (4)	56 (10)	65 (5)	4.1
67	Imazethapyr	0.9999	1	0.3	67 (4)	63 (6)	71 (10)	73 (11)	53 (21)	76 (7)	76 (8)	62 (2)	-11.1
68	Imidacloprid	0.9985	1	0.3	73 (9)	72 (8)	70 (12)	73 (7)	73 (17)	70 (11)	74 (3)	76 (5)	-29.5
69	Iprovalicarb	0.9998	1	0.3	70 (10)	65 (10)	75 (11)	74 (13)	63 (12)	66 (9)	70 (3)	73 (4)	27.6
70	Linuron	0.9999	1	0.3	105 (7)	94 (9)	92 (3)	90 (4)	90 (12)	95 (10)	99 (5)	102 (4)	40.9
71	Malathion	0.9965	1	0.3	73 (18)	83 (6)	91 (5)	93 (5)	71 (14)	82 (8)	98 (7)	101 (3)	48.2
72	Mecarbam	0.9963	1	0.3	88 (16)	74 (6)	88 (10)	93 (7)	85 (11)	86 (12)	105 (7)	105 (4)	56.2
73	Mephosfolan	0.9948	1	0.3	70 (9)	76 (14)	97 (11)	99 (5)	70 (14)	80 (5)	100 (5)	107 (2)	6.5
74	Mepronil	0.9961	1	0.3	72 (14)	79 (10)	102 (7)	105 (6)	70 (12)	80 (8)	105 (5)	90 (2)	44.9
75	Metalaxyl	0.9993	1	0.3	95 (5)	90 (9)	92 (7)	95 (7)	77 (9)	88 (3)	95 (6)	100 (3)	10.4
76	Metconazole	0.9962	5	1.5	72 (10)	83 (9)	94 (7)	97 (4)	70 (12)	81 (7)	102 (5)	104 (2)	40.7
77	Methidathion	0.9986	1	0.3	78 (3)	88 (5)	89 (4)	92 (4)	82 (5)	89 (5)	100 (4)	107 (5)	18.9
78	Methiocarb	0.9995	1	0.3	59 (54)	77 (10)	97 (12)	97 (11)	80 (38)	95 (20)	90 (2)	80 (3)	49.5
79	Methiocarb sulfone	0.9980	1	0.3	82 (9)	84 (10)	86 (6)	90 (8)	71 (10)	80 (8)	97 (6)	100 (4)	5.8
80	Methoxyfenozide	0.9977	1	0.3	71 (11)	82 (14)	87 (10)	88 (8)	79 (11)	81 (11)	99 (7)	104 (7)	85.3
81	Metsulfuron-methyl	0.9994	1	0.3	84 (3)	93 (3)	98 (5)	101 (4)	90 (9)	92 (5)	95 (4)	95 (2)	-24.3
82	Metribuzin	0.9991	1	0.3	89 (43)	89 (18)	100 (19)	90 (9)	71 (28)	86 (18)	105 (17)	107 (12)	13.5
83	Mevinphos	0.9999	1	0.3	87 (16)	80 (12)	79 (9)	80 (8)	97 (16)	97 (8)	96 (3)	97 (1)	6.4
84	Myclobutanil	0.9998	5	1.5	111 (24)	83 (37)	75 (17)	91 (17)	71 (4)	88 (29)	89 (16)	92 (15)	7.4
85	Molinate	0.9996	1	0.3	93 (4)	95 (6)	96 (3)	97 (4)	88 (12)	89 (5)	96 (3)	97 (1)	27.7
86	Monocrotophos	0.9957	1	0.3	56 (24)	87 (14)	98 (8)	103 (5)	78 (22)	87 (13)	88 (10)	96 (3)	-8.5
87	Monolinuron	0.9934	1	0.3	72 (30)	77 (19)	85 (11)	82 (9)	64 (30)	74 (18)	89 (16)	98 (6)	18.2
88	Nicosulfuron	0.9995	1	0.3	99 (19)	100 (4)	95 (8)	97 (3)	82 (11)	90 (8)	88 (10)	96 (3)	-23.3
89	Nuarimol	0.9973	1	0.3	71 (16)	85 (11)	93 (5)	98 (8)	87 (15)	85 (5)	99 (7)	98 (4)	9.8
90	Oxadixyl	0.9997	1	0.3	76 (6)	72 (10)	72 (7)	72 (6)	87 (8)	93 (7)	91 (5)	91 (6)	3.4
91	Oxamyl	0.9929	1	0.3	110 (31)	77 (30)	79 (18)	84 (14)	91 (43)	84 (34)	95 (19)	98 (10)	-21.5
92	Ethyl paraoxon	0.9945	1	0.3	65 (55)	56 (30)	73 (14)	81 (17)	117 (36)	93 (24)	100 (14)	100 (15)	115.5
93	Pencycuron	0.9994	1	0.3	114 (5)	97 (5)	93 (16)	94 (5)	85 (5)	110 (42)	109 (10)	98 (14)	82.7
94	Penconazole	0.9990	1	0.3	71 (9)	84 (9)	96 (9)	98 (7)	97 (12)	92 (9)	101 (10)	98 (4)	70.3
95	Penoxsulam	0.9952	5	1.5	59 (18)	78 (10)	88 (14)	90 (11)	50 (21)	73 (4)	94 (9)	107 (3)	-58.1
96	Picoxystrobin	0.9993	1	0.3	78 (21)	85 (8)	88 (14)	87 (13)	82 (12)	75 (14)	95 (6)	99 (6)	173.6
97	Pyraclostrobin	0.9996	1	0.3	104 (17)	88 (16)	86 (18)	80 (15)	87 (18)	86 (23)	109 (20)	109 (12)	61.7

Nr.	Compounds	r^2	LOQ µg kg⁻¹	LOD µg kg⁻¹	Recovery (%) (Precision, RSD_R %) Spiking levels (µg kg⁻¹), n = 7				Recovery (%) (RSD_{IP} %) Spiking levels (µg kg⁻¹), n = 7				Matrix effect (%)
					1	5	10	20	1	5	10	20	
98	Pyrazophos	0.9963	1	0.3	114 (12)	103 (19)	89 (7)	78 (20)	85 (14)	84 (8)	96 (6)	107 (7)	-23.1
99	Pyridaphenthion	0.9967	1	0.3	73 (17)	86 (11)	99 (8)	102 (6)	70 (19)	78 (13)	98 (4)	107 (3)	18.4
100	Pyrimethanil	0.9975	1	0.3	70 (4)	82 (8)	91 (6)	94 (10)	71 (5)	75 (12)	79 (7)	82 (4)	74.6
101	Pirimicarb	0.9964	1	0.3	78 (5)	79 (7)	94 (6)	100 (4)	75 (15)	85 (8)	94 (4)	96 (2)	15.8
102	Ethyl pirimiphos	0.9954	5	1.5	46 (28)	58 (10)	70 (8)	71 (5)	62 (18)	57 (13)	79 (7)	82 (4)	191.5
103	Methyl pirimiphos	0.9994	1	0.3	89 (3)	91 (6)	94 (8)	96 (10)	87 (6)	89 (2)	102 (5)	111 (2)	104.2
104	Pyriproxyfen	0.9964	5	1.5	61 (12)	78 (8)	83 (9)	82 (9)	57 (8)	75 (8)	96 (10)	113 (2)	439.8
105	Procymidone	0.9972	1	0.3	71 (16)	79 (8)	100 (12)	104 (9)	71 (18)	90 (7)	102 (5)	111 (2)	153.2
106	Propanil	0.9938	1	0.3	55 (23)	81 (11)	77 (18)	71 (12)	40 (3)	70 (3)	95 (12)	117 (4)	58.8
107	Prometryn	0.9929	1	0.3	107 (9)	89 (18)	91 (4)	76 (19)	82 (23)	88 (16)	92 (8)	107 (5)	131.5
108	Quinalphos	0.9997	1	0.3	76 (44)	82 (16)	85 (20)	90 (8)	80 (42)	89 (20)	103 (12)	102 (9)	111.3
109	Quizalofop-P-ethyl	0.9964	5	1.5	60 (29)	62 (11)	75 (10)	77 (6)	37 (29)	55 (15)	81 (6)	87 (4)	166.3
110	Saflufenacil	0.9968	1	0.3	119 (10)	89 (20)	83 (15)	76 (16)	77 (31)	90 (16)	88 (10)	85 (10)	-35.7
111	Simazine	0.9956	5	1.5	59 (19)	70 (16)	83 (14)	87 (8)	53 (17)	71 (8)	91 (6)	104 (3)	46.0
112	Tebuconazole	0.9996	1	0.3	89 (5)	84 (12)	85 (13)	86 (9)	101 (8)	104 (4)	103 (5)	105 (2)	35.4
113	Tebufenozide	0.9972	1	0.3	79 (43)	85 (15)	84 (12)	94 (11)	83 (26)	87 (16)	90 (10)	86 (8)	152.1
114	Tebufenpyrad	0.9952	5	1.5	55 (28)	72 (18)	89 (7)	93 (9)	68 (14)	75 (13)	95 (6)	98 (3)	129.4
115	Tetraconazole	0.9942	5	1.5	157 (14)	119 (29)	89 (8)	78 (17)	68 (28)	79 (34)	105 (17)	104 (14)	46.3
116	Thiabendazole	0.9996	1	0.3	87 (26)	74 (18)	82 (20)	74 (9)	47 (105)	53 (23)	88 (22)	107 (5)	-4.2
117	Thiacloprid	0.9981	1	0.3	83 (10)	87 (11)	97 (14)	103 (5)	73 (19)	85 (15)	94 (4)	94 (2)	3.6
118	Thiamethoxam	0.9999	1	0.3	78 (4)	76 (3)	74 (6)	77 (5)	77 (7)	74 (6)	72 (3)	79 (4)	-19.6
119	Thiobencarb	0.9986	1	0.3	81 (4)	91 (3)	97 (5)	100 (5)	83 (5)	89 (4)	94 (4)	94 (2)	104.2
120	Thiophanate-methyl	0.9999	1	0.3	85 (4)	86 (11)	91 (5)	93 (3)	85 (15)	88 (11)	98 (7)	98 (8)	4.0
121	Triazophos	0.9989	1	0.3	86 (14)	87 (16)	95 (13)	93 (11)	47 (12)	63 (8)	95 (6)	107 (3)	30.2
122	Triadimefon	0.9985	1	0.3	73 (2)	73 (14)	84 (13)	84 (7)	107 (14)	101 (10)	105 (6)	114 (5)	6.3
123	Triadimenol	0.9975	1	0.3	74 (6)	85 (6)	95 (4)	99 (4)	82 (4)	86 (4)	95 (7)	104 (6)	-6.9
124	Tricyclazole	0.9990	1	0.3	78 (20)	87 (15)	93 (16)	92 (5)	84 (19)	86 (8)	103 (9)	103 (6)	18.0
125	Trichlorfon	0.9952	5	1.5	59 (68)	72 (15)	85 (15)	91 (7)	108 (23)	83 (13)	90 (7)	93 (1)	-7.8
126	Trifloxystrobin	0.9992	1	0.3	77 (5)	75 (10)	80 (10)	81 (11)	79 (8)	76 (5)	79 (6)	85 (4)	76.3
127	Triflusulfuron	0.9995	1	0.3	75 (28)	80 (12)	88 (6)	88 (5)	98 (38)	89 (12)	90 (7)	93 (1)	-48.9
128	Triflumizole	0.9959	1	0.3	72 (19)	77 (9)	83 (7)	85 (6)	72 (30)	78 (11)	94 (3)	97 (5)	128.3
129	Triflumuron	0.9999	1	0.3	84 (44)	85 (14)	86 (9)	84 (7)	122 (13)	114 (5)	100 (8)	103 (17)	88.8
130	Vamidothion	0.9947	1	0.3	71 (13)	80 (6)	88 (9)	91 (8)	73 (16)	75 (10)	94 (3)	97 (5)	-8.2

Table S3. Comparison of sample preparation and analysis methods for pesticide residues in fish fillets with their respective limits of quantification (LOQ)

Compounds	Sample Preparation		Analytical technique	LOQ (μg kg^{-1})	Reference
	Extraction	Clean-up and/or concentration			
182 contaminants (pesticides and veterinary drugs)	10 g sample + 10 mL MeCN → shake for 1 min → 4 g Na_2SO_4 and 1 g NaCl → shake by hand → centrifugation	1 mL supernatant → 150 mg Na_2SO_4 and the sorbents PSA 25 mg and C18 125 mg → diluted 5× in ultrapure water	LC-QTOFMS	0.15-3.6	33
340 pesticides	10 g sample + 10 mL MeCN with 1% formic acid → manual shaking 1 min + 4 g $MgSO_4$ + 1 g NaCl + 1 g Na_3Cit + 0.5 g $Na_3Cit{\cdot}1.5H_2O$ + 1 g chitin → shaken 1 min → centrifugation 4000 rpm 10 min	no clean-up	UHPLC-MS/MS	0.16-4	47
30 pesticides	10 g sample + 10 mL MeCN + 1.5 g NaCl and 4.0 g MgSO4 → vortex mixing for 4 min → centrifugation at 3500 ×g for 5 min	7 mL supernatant + 350 mg PSA, 180 mg C18 and 1000 mg $MgSO_4$ → vortex mixing for 1 min → centrifugation at 3500 ×g for 5 min	UHPLC-MS/MS	1.0–15.0	48
52 highly polar pesticides	10 g sample + 10 mL MeCN → 4 g MgSO4, 1 g NaCl, 1 g Na_3Cit, and 0.5 g $Na_3Cit{\cdot}1.5H_2O$ → vortex mixing for 1 min → centrifugation 4000 rpm for 5 min	7 mL supernatant + 400 mg PSA, 400 mg C18 and 1200 mg $MgSO_4$ → vortex mixing for 1 min → centrifugation at 4000 rpm for 5 min	UHPLC-MS/MS	1-10	49
77 pesticides	10 g sample + 10 mL MeCN → agitation for 1 min → 4 g $MgSO_4$ + 1.5 g NaCl → vortex 5 min → centrifugation at 2260 ×g for 5 min	7 mL supernatant + 1 g $MgSO_4$ + 350 mg PSA + 180 mg C18 → vortex mixing for 1 min → centrifugation at 2260 ×g for 5 min	UHPLC-MS/MS	2.23	44
Pyraclostrobin	2 g sample + 5 mL MeCN → shaking for 4 min → 1 g NaCl + 0.5 g $MgSO_4$ → shaking 3 min → centrifugation at 1425 ×g for 5 min	1.5 mL supernatant + 500 mg $MgSO_4$ + 200 mg C18 + 200 mg PSA	UHPLC-MS/MS	5-25	50
302 pesticides	4 g sample + 4 mL MeCN + shaking 1 min → 2 g $MgSO_4$ + NaCl (4:1 w/w) → shaker for 10 min → centrifugation at 4150 rpm for 3 min	no clean-up	UHPLC-MS/MS	5–40	51
66 pesticides (insecticides, fungicides, and herbicides)	2 g sample + 1 mL 50 mM ammonium acetate in 0.1 M Na_2EDTA → vortex 1 min → 9 mL MeCN + 500 mg NaAc → vortex 3 min → centrifugation at 4500 ×g for 10 min	5 mL supernatant → 200 mg PSA and 300 mg C18 → vortex 1 min → centrifuge at 4500 ×g for 10 min	LC-MS/MS	10	16

Compounds	Sample Preparation		Analytical technique	LOQ (μg kg^{-1})	Reference
	Extraction	Clean-up and/or concentration			
9 herbicides	5 g sample + 5 mL H_2O + 10 mL MeCN containing 1% formic acid + 2 g NaCl and 5 g $MgSO_4$ → vortex 1 min → ultrasonic bath 10 min → centrifugation at 5000 rpm for 5 min	0.5 g neutral alumina and 0.5 g $MgSO_4$ → shaking 1 min → centrifugation at 5000 rpm for 5 min → evaporation → redissolved in 1 mL of methanol:5 mM NH_4Ac (1:1) solution	UHPLC-MS/MS	0.5-1.5	52
22 pesticides	2 g sample + 4 mL MeCN with 1% acetic acid → vortex for 10 min → 4 g $MgSO_4$ + 0.1 g NaCl → vortex for 5 min → centrifugation at 8000 rpm for 5 min	Method 1 (SPE): 2 mL supernatant → PEP Plus column → elution with 1.2 mL MeCN → evaporation (N_2) → redissolve in 1.0 mL MeCN; Method 2 (SPE - d-SPE): 2 mL supernatant → PEP Plus column → 1.2 mL MeCN → evaporation (N_2) → redissolve in 1.0 mL MeCN → 2 mL + 30 mg PSA + 60 mg C18 + 100 mg $MgSO_4$ → centrifugation at 5000 rpm for 5 min	UHPLC-MS/MS	0.5–5	53
25 pesticides	10 g sample + 10 mL MeCN + vortex 1 min + 4 g $MgSO_4$ + 1 g NaCl → centrifugation 5000 rpm for 5 min	2 mL + 50 mg PSA + 50 mg GCB + 150 mg $MgSO_4$ → centrifugation 10,000 rpm → evaporation N_2 → redissolution 1 mL toluene	UHPLC-MS/MS	120-170	54
110 pesticides and 2 metabolites	5 g sample + 5 mL H_2O + 10 mL MeCN + ultrasonication 15 min → 1.5 g NaCl + 4.0 g $MgSO_4$ → shake 1 min → centrifugation at 8000 rpm for 3 min	9 mL supernatant → 120 mg Poly-MDN + 10 mg C18 + 10 mg GCB → shake for 30 s → external magnet for 3 s → transfer the solution → dry with N_2 → reconstitute in 0.9 mL	UHPLC-MS/MS	0.1–2	55
161 pesticides, 63 veterinary drugs, 24 metabolites, and 14 environmental contaminants	2 g sample + 10 mL 4:1 (v/v) mixture of MeCN:water -> vortex for 10 min + centrifugation at 3711 ×g for 3 min	204 μL supernatant → evaporation (N_2 at 40 °C) + redissolution (731.8 μL water + 18.2 μL MeCN) → centrifugation 13,000 ×g for 5 min	UHPLC-MS/MS		56
Herbicides and metabolites	2 g sample + 5 mL MeCN with 0.1% formic acid (pH 3) → vortex mixing for 5 min → 3 g NaCl + shake for 2 min	1.5 mL supernatant + 150 mg $MgSO_4$ + 50 mg C18 → vortex 1 min → centrifuge at 8000 rpm for 2 min	UHPLC-MS/MS	0.02–6.35	57
90 pesticides	2 g sample + 4 mL MeCN with 0.2% formic acid → vortex at 2500 rpm for 10 min + 0.4 g $MgSO_4$ + 0.1 g NaCl → vortex for 5 min → centrifugation at 8000 rpm for 5 min	2 mL supernatant → Prime HLB cartridge	UHPLC-MS/MS	0.05–5	58

Compounds	Sample Preparation		Analytical technique	LOQ (μg kg^{-1})	Reference
	Extraction	Clean-up and/or concentration			
32 pesticides	1 g sample + 10 mL H_2O + 10 mL MeCN with 1% ammonium formate → vortex 1 min → 4 g $MgSO_4$ + 0.5 g Na_3Cit + 0.5 g $Na_3Cit{\cdot}1.5H_2O$ → vortex 1 min → centrifugation at 4500 rpm for 10 min	10 mL supernatant + 400 mg PSA + C18 and 1200 mg MgSO4 → vortex 1 min → centrifugation at 4500 rpm for 10 min → evaporation of supernatant → reconstitution to 1 mL → H_2O:MeCN (90:10) → vortex 1 min	LC-MS/MS	0.5–5	46
57 pesticides	5 g sample + 20 mL MeCN → shake 20 min → 4 g $MgSO_4$ + 1 g NaCl + 1 g Na_3Cit + 0.5 g $Na_3Cit{\cdot}1.5H_2O$ → shake 1 min → centrifugation at 4000 rpm for 5 min	1 mL supernatant → 150 mg $MgSO_4$ + 100 mg PSA + 100 mg C18 → shake for 1 min → centrifuge at 4000 rpm for 3 min	GC-MS/MS	3–7	45
263 pesticides + metabolites	5 g sample + 10 mL MeCN → shake 1 min → 4 g $MgSO_4$ + 1 g NaCl → shake 10 min → centrifugation at 4500 ×g for 10 min	1 mL supernatant + 150 mg $MgSO_4$+ 25 mg PSA + 25 mg C18 → shake 1 min → centrifuge at 13,000 ×g for 10 min	LC-MS/MS<br>GC-MS/MS	10	42
95 pesticides and veterinary drugs	2.5 g sample + 10 mL MeCN → shake 10 min + 2.0 g Na_2SO_4 + 100 μL acetic acid → centrifugation at 5856 ×g for 10 min	chitosan microspheres (60 mg) in a clean-up column + 2.0 mL extract → vortex 90 min → extract expelled from the column, and transferred to a 2 mL centrifuge tube	UHPLC-MS/MS	1–2	59
63 pesticides	2.5 g sample + 0.5 mL Na_2SO_4 + 10 mL MeCN → vortex 10 min + 2.0 g Na_2SO_4 + 100 μL acetic acid → centrifugation 8000 ×g 10 min	Clean-up by semi-automatic centrifugation using a vegetable sponge cartridge (50 mg)	UHPLC-MS/MS	1–2	37
130 pesticides	5 g sample + 10 mL MeCN with 0.1% (v/v) formic acid → vortex 1 min → 4 g $MgSO_4$ + 1 g NaCl → vortex 1 min → centrifugation at 4850 ×g for 8 min	1 mL supernatant + 100 mg of chitosan-modified vegetable sponge (CMVS) → vortex 1 min → centrifugation at 4700 ×g for 6 min	UHPLC-MS/MS	1	This work

C18: octadecylsilane; Na_2EDTA: ethylenediaminetetraacetic acid disodium salt; GC-MS/MS: gas chromatography with tandem mass spectrometry; LC-MS/MS: liquid chromatography with tandem mass spectrometry; LC-QTOFMS: liquid chromatography with quadrupole time-of-flight mass spectrometry; MeCN: acetonitrile; $MgSO_4$: magnesium sulfate; NaAc: sodium acetate; Na_2SO_4: sodium sulfate; Na_3Cit: sodium citrate; $Na_3Cit{\cdot}1.5H_2O$: disodium citrate sesquihydrate; NH_4Ac: ammonium acetate; PSA: primary-secondary amine; r-DSPE: reversed-dispersive solid-phase extraction; UHPLC-MS/MS: ultra-high performance liquid chromatography with tandem mass spectrometry.
